# Editorial: Exploring precision medicine in reconstructive and aesthetic surgery

**DOI:** 10.3389/fsurg.2026.1902078

**Published:** 2026-07-10

**Authors:** Domenico Tripodi, Mario Faenza, Claudio Cannistra

**Affiliations:** 1 Department of Medicine, Saint Camillus International University of Health and Medical Sciences, Rome, Italy; 2 Multidisciplinary Department of Medical, Surgical and Dental Sciences, University of Campania “Luigi Vanvitelli”, Caserta, Italy; 3 Department of Surgery, Hôpital Bichat-Claude Bernard, Paris, France

**Keywords:** personalized medicine, plastic surgery, precision medicine, reconstructive surgical procedures, surgery

## Introduction

Precision medicine is increasingly influencing surgical disciplines by promoting patient-centred, data-informed, and anatomy-driven decision-making. In reconstructive and aesthetic surgery, where interindividual variability in anatomy, tissue characteristics, comorbidities, and patient expectations is substantial, the adoption of precision-based approaches is particularly relevant. The *Research Topic* “Exploring Precision Medicine in Reconstructive and Aesthetic Surgery” was conceived to capture how personalised strategies ranging from surgical planning and intraoperative techniques to outcome prediction and perioperative management—are shaping contemporary practice. The *Research Topic* brings together 12 contributions spanning reconstructive, aesthetic, oncologic, and perioperative domains. Collectively, these articles illustrate how precision medicine can enhance safety, reproducibility, and clinical outcomes by integrating anatomical knowledge, quantitative assessment tools, imaging guidance, and biologically informed decision-making.

## Standardisation and anatomy-driven personalisation in aesthetic surgery

One of the key pillars of precision medicine is the standardisation of techniques grounded in anatomical precision while allowing flexibility for individual patient characteristics. This concept is exemplified by the expert consensus on facial embedded thread lift techniques, representing the first structured national guideline developed by Chinese experts (Shi). By integrating operator stratification, material selection, and region-specific anatomical considerations, this consensus addresses the variability that has historically limited reproducibility and safety in minimally invasive aesthetic procedures. Complementing this approach, advances in computational modelling further support personalised aesthetic assessment. The application of a multi-objective genetic algorithm for facial landmark modelling demonstrates how medical aesthetic principles can be translated into explainable, data-driven tools aligned with expert clinical judgement (Ye et al.). Such approaches highlight the growing role of artificial intelligence in supporting objective yet personalised aesthetic evaluation.

## Quantitative outcome assessment and digital precision

Accurate outcome assessment is essential for the implementation of precision medicine. Digital anthropometry has emerged as a valuable tool for documenting surgical results and supporting patient engagement. The study on post-bariatric body contouring surgery demonstrates how three-dimensional avatars and clinimetric assessments can objectively quantify anatomical changes and correlate them with patient-reported outcomes after abdominoplasty and thighplasty (Minetto et al.). Similarly, the investigation of tension-reducing suture techniques in maxillofacial plastic surgery highlights how personalised wound-closure strategies can significantly influence complication rates, scar quality, and patient satisfaction (Du et al.). Together, these contributions underscore the importance of quantitative, reproducible outcome measures in precision-driven surgical care.

## Precision reconstructive strategies and flap optimisation

Reconstructive surgery inherently requires tailored approaches based on defect characteristics and patient-specific anatomy. Several contributions in this *Research Topic* address flap optimisation and reconstructive planning. The comprehensive review of the temporalis muscle flap provides an updated overview of anatomical considerations, surgical refinements, and emerging technologies such as virtual surgical planning and minimally invasive approaches (Landfald et al.). In parallel, the long-term follow-up study on a modified shunt-restricted instep arterialised venous flap for hand reconstruction demonstrates how precise vascular modifications can improve flap survival, aesthetic integration, and functional recovery over extended follow-up periods (Chen et al.). These studies exemplify how classical reconstructive techniques can be refined through anatomical insight and outcome-oriented innovation.

## Personalised perioperative and oncologic surgical care

Precision medicine also extends to perioperative management and oncologic planning. Ultrasound-guided regional anaesthesia represents a clear example of image-guided personalisation, particularly in elderly patients. By enabling targeted nerve blocks and reducing systemic drug exposure, ultrasound-guided techniques support safer anaesthetic management and enhanced recovery pathways (Lin et al.). In breast surgery, anatomy-based and imaging-assisted strategies further illustrate precision-oriented care. The analysis of preshaping procedures in prophylactic nipple-sparing mastectomy demonstrates how anatomical optimisation can reduce complications in high-risk patients with challenging breast morphology (Maciulaitis et al.). Similarly, the ultrasound-assisted periareolar oncoplastic approach highlights how intraoperative imaging can improve surgical precision, cosmetic outcomes, and patient satisfaction in breast-conserving surgery (De Luca et al.).

## Biomarkers, biology, and personalised risk stratification

Several contributions emphasise the role of biological and clinical markers in tailoring surgical care. The identification of body mass index and neutrophil-to-lymphocyte ratio as independent predictors of postoperative drainage volume after reduction mammoplasty provides a practical framework for personalised postoperative management (Wu et al.). Likewise, the study on modified nerve decompression combined with murine nerve growth factor therapy for diabetic peripheral neuropathy highlights how targeted anatomical intervention combined with biological augmentation may enhance clinical outcomes (Zhang et al.). Finally, the narrative review on the impact of sex hormones on postoperative recovery synthesises evidence on the biological mechanisms influencing wound healing, angiogenesis, and inflammation, reinforcing the importance of hormonally informed and sex-specific approaches to personalised surgical care (Lv et al.).

## Conclusions and future perspectives

The contributions collected in this *Research Topic* demonstrate that precision medicine in reconstructive and aesthetic surgery is a multidimensional paradigm encompassing anatomical precision, quantitative outcome assessment, imaging guidance, biomarker-driven stratification, and patient-centred decision-making. While many of the presented approaches show promising clinical value, further multicentre validation and standardisation will be essential to support widespread implementation. As data integration, artificial intelligence, and personalised biological markers continue to evolve, reconstructive and aesthetic surgery is well positioned to benefit from precision-driven innovation. We hope that this *Research Topic* will stimulate further interdisciplinary collaboration and contribute to safer, more predictable, and more individualised surgical care.

